# Fractalkine is a “find-me” signal released by neurons undergoing ethanol-induced apoptosis

**DOI:** 10.3389/fncel.2014.00360

**Published:** 2014-11-07

**Authors:** Jennifer D. Sokolowski, Chloe N. Chabanon-Hicks, Claudia Z. Han, Daniel S. Heffron, James W. Mandell

**Affiliations:** ^1^Department of Pathology, School of Medicine, University of VirginiaCharlottesville, VA, USA; ^2^Medical Scientist Training Program, School of Medicine, University of VirginiaCharlottesville, VA, USA; ^3^Neuroscience Graduate Program, School of Medicine, University of VirginiaCharlottesville, VA, USA; ^4^Department of Microbiology, Immunology and Cancer Biology, School of Medicine, University of VirginiaCharlottesville, VA, USA; ^5^Center for Cell Clearance, University of VirginiaCharlottesville, VA, USA

**Keywords:** apoptosis, fractalkine, ethanol, cytokine, chemokine, neuron, microglia, clearance

## Abstract

Apoptotic neurons generated during normal brain development or secondary to pathologic insults are efficiently cleared from the central nervous system. Several soluble factors, including nucleotides, cytokines, and chemokines are released from injured neurons, signaling microglia to find and clear debris. One such chemokine that serves as a neuronal–microglial communication factor is fractalkine, with roles demonstrated in several models of adult neurological disorders. Lacking, however, are studies investigating roles for fractalkine in perinatal brain injury, an important clinical problem with no effective therapies. We used a well-characterized mouse model of ethanol-induced apoptosis to assess the role of fractalkine in neuronal–microglial signaling. Quantification of apoptotic debris in fractalkine-knockout (KO) and CX3CR1-KO mice following ethanol treatment revealed increased apoptotic bodies compared to wild type mice. Ethanol-induced injury led to release of soluble, extracellular fractalkine. The extracellular media harvested from apoptotic brains induces microglial migration in a fractalkine-dependent manner that is prevented by neutralization of fractalkine with a blocking antibody or by deficiency in the receptor, CX3CR1. This suggests fractalkine acts as a “find-me” signal, recruiting microglial processes toward apoptotic cells to promote their clearance. Next, we aimed to determine whether there are downstream alterations in cytokine gene expression due to fractalkine signaling. We examined mRNA expression in fractalkine-KO and CX3CR1-KO mice after alcohol-induced apoptosis and found differences in cytokine production in the brains of these KOs by 6 h after ethanol treatment. Collectively, this suggests that fractalkine acts as a “find me” signal released by apoptotic neurons, and subsequently plays a critical role in modulating both clearance and inflammatory cytokine gene expression after ethanol-induced apoptosis.

## INTRODUCTION

Clearance of apoptotic neurons is critical for normal central nervous system (CNS) development and for resolution of injury due to pathologic processes. Failure to clear apoptotic neurons leads to secondary necrosis with leakage of intracellular contents that are toxic and inflammatory. Efficient clearance is thought to be critical in order to avoid an adverse immune reaction and secondary degeneration (Ravichandran and Lorenz, 2007). However, the precise mechanisms by which apoptotic neurons are cleared have yet to be elucidated. Most of the work on apoptotic cell clearance has been done in non-neural systems or in invertebrate animal models (Truman et al., 2008; Elliott et al., 2009; Gronski et al., 2009). Elucidation of molecular mechanisms used for clearance of apoptotic cells in the developing and adult mammalian brain is an important goal.

Studies in peripheral tissues have shown that as cells undergo apoptosis, they release soluble signals that attract phagocytes and modulate their clearance (Ravichandran and Lorenz, 2007), and the chemokine fractalkine has been described as one of many “find me” signals released by apoptotic cells (Truman et al., 2008). Fractalkine is a transmembrane chemokine that is cleaved constitutively by matrix metalloprotease ADAM10 and inducibly by ADAM 17 (also known as the TNFα-converting enzyme, TACE) to release an extracellular soluble fragment. Inducible cleavage occurs following cell stress or injury, and the soluble fragment acts as a chemotactic factor for T cells, monocytes, and microglia (Bazan et al., 1997; Harrison et al., 1998). The study describing fractalkine as a “find me” signal showed that it is released following induction of apoptosis and that the fractalkine receptor, CX3CR1, modulates recruitment of phagocytes to apoptotic germinal center B cells (Truman et al., 2008). In this study we asked whether fractalkine and its receptor, CX3CR1 are important for apoptotic neuron clearance *in vivo* utilizing a mouse model of fetal alcohol syndrome.

In the CNS, fractalkine is expressed by neurons and cleaved by matrix metalloproteases to release a soluble fragment after neuronal stress. Microglia are the only CNS cell that expresses appreciable levels of the fractalkine receptor, CX3CR1 *in vivo* (Harrison et al., 1998). Studies in CNS models have largely focused on the role of fractalkine in neurotoxicity and have shown that fractalkine signaling modulates the inflammatory response of microglia (Mizuno et al., 2003; Noda et al., 2011). However, whether fractalkine signaling promotes a beneficial versus a detrimental response has been unclear, as studies have come to different conclusions depending on the injury model and the outcomes measured (Mizuno et al., 2003; Cardona et al., 2006; Fuller and Van Eldik, 2008; Staniland et al., 2010; Noda et al., 2011).

Other studies in the CNS have also shown that fractalkine signaling plays a role in developmental pruning of neurons. Mice lacking CX3CR1 have increased density of dendritic spines (Paolicelli et al., 2011), and CX3CR1 deficiency leads to delayed development of the barrel cortex (Hoshiko et al., 2012). There are corollaries between events that occur during pruning and events that occur in neuronal degeneration after apoptosis.

A well-characterized mouse model of ethanol-induced injury has proven very useful for studying developmental neuronal apoptotic mechanisms. Ethanol injection at postnatal day 7 causes robust forebrain neuronal apoptosis (as opposed to other forms of cell death such as necrosis) and the dose required and time course have been well-characterized (Ikonomidou et al., 2000). This model has been extensively used to study factors involved in the neuronal apoptotic cascade (Young et al., 2003; Ghosh et al., 2009). However, this model has not been employed to assess what factors may be involved in orchestrating clearance of apoptotic neurons or the response of neighboring glia.

Our data provide strong evidence of a role for fractalkine signaling in the response to acute alcohol neurotoxicity. We show that deficiency in fractalkine or the receptor leads to increased apoptotic debris and an altered inflammatory reaction after ethanol-induced apoptosis. Our experiments suggest that fractalkine release from apoptotic neurons may act as a “find me” signal to modulate the microglial response and promote clearance.

## MATERIALS AND METHODS

### MICE

All animal procedures were approved by the University of Virginia Animal Care and Use Committee. Mice used were C57/bl6 (Charles River), CX3CR1^eGFP/+^ and CX3CR1^eGFP/eGFP^ (Jung et al., 2000) or fractalkine-knockout (KO; Cook et al., 2001) on C57/bl6 background. CX3CR1^eGFP^ mice have green fluorescent protein (GFP) inserted into the CX3CR1 locus, therefore CX3CR1^eGFP/eGFP^ animals are functional KOs.

### ETHANOL INJURY

Ethanol was injected subcutaneously in postnatal day 7 pups as a 20% solution in 0.9% saline at 15.9 μL/g body weight. Control animals were injected with 0.9% saline at 15.9 μL/g body weight. It was administered twice, 2 h apart (as described by Ghosh et al., 2009).

### IMMUNOSTAINING AND QUANTIFICATION OF APOPTOTIC DEBRIS

We quantified the number of apoptotic corpses in the cortex in ethanol-treated and control-treated animals. We used CX3CR1^eGFP/eGFP^, fractalkine-KO, and wild type (WT) mice. We used 3–6 animals per condition. Brain tissue was harvested 4 or 6 h after the first injection and was fixed in either 4% paraformaldehyde or 70% ethanol. Anti-fractin, an antibody against caspase-cleaved actin, is a sensitive and specific marker of apoptotic neuronal debris (Suurmeijer et al., 1999; Sokolowski et al., 2014), therefore we quantified apoptosis by counting the number of fractin-stained corpses. For fractin staining, tissue was processed into paraffin by standard methods. Paraffin-embedded sections were dewaxed, rehydrated, underwent antigen retrieval (Tris-EDTA pH 9, 12 min over a boiling water bath), were quenched (15 min, 0.6% H_2_O_2_ in dH_2_O) and blocked (1 h, 2% horse serum, 0.1% Tween in PBS) prior to incubation with primary antibody (overnight at 4°C, diluted in block), fractin (Millipore, 1:1000). Immunoperoxidase detection was performed using the ImmPress polymeric peroxidase reagents (Vector). Diaminobenzidine (Dako) 1 mg/ml plus 0.02% hydrogen peroxide was applied for 3–5 min. We quantified apoptosis by counting the number of fractin-stained corpses in the cortex and hippocampus. We averaged the number of corpses in three sections per animal, and quantified 3–6 animals per condition. Brightfield images were acquired with an Olympus BX40 upright microscope and a Scion Firewire CCD camera (Scion, Frederick, MD, USA).

### BRAIN-CONDITIONED MEDIA

Brain-conditioned media (BCM) was prepared from WT mice as follows: brains harvested at 6 h after ethanol or saline treatments were hemisected and three coronal cuts were made. These tissue chunks were incubated in DMEM (no antibiotics, 1 mL per brain) in a 15 mL conical tube for 2 h on ice, on a rocker. The media was then isolated, excluding the tissue, and filtered through a 0.4 μm filter and stored at –20°C until use.

### WESTERN BLOTTING

Brain-conditioned media from 2 brains was collected and a BCA protein assay (Pierce) was performed. Protein (2 mg) was precipitated in 15% TCA at 4°C for 2 h. The precipitate was spun down at 12 k rpm for 15 min at 4°C. The resulting pellet was washed three times with ice-cold acetone. The pellet was resuspended in 2X alkaline sample buffer (100 mM Tris pH 8.0, 4% SDS, 200 mM DTT, 20% glycerol). A NuPAGE gel (Life Technologies) was loaded with 50 μg of protein per lane and separated by electrophoresis using standard procedures. Gels were transferred to a PVDF membrane (Immobilon) for 90 min with a semidry transfer apparatus and treated with blocking reagent (LI-COR block; LI-COR, Lincoln NE) for 1 h and then probed with primary antibodies overnight. Antibodies used were the following: rat monoclonal anti-fractalkine (1:500, R&D systems). For visualization, blots were incubated with fluorescent secondary antibodies (1:2000, LI-COR) for 2 h and imaged on a LI-COR Odyssey infrared scanner.

### CELL CULTURE

Glia were harvested from the forebrain of newborn pups (postnatal day 1–3). Briefly, meninges were removed from the brain and tissue was dissociated in 0.05% trypsin EDTA for 10 min at 37°C. Following trituration, cells were suspended in DMEM supplemented with 10% fetal bovine serum and plated into flasks. Cells were grown in an incubator at 37°C, 5% CO_2_. Media was replaced twice per week for 2 weeks to obtain mixed glial culture. To harvest glia-conditioned media, media on mixed glial cultures was changed to fresh growth media (DMEM with 10% FBS), then this media was collected after 24–48 h and filtered through a 0.4 μm filter and used immediately. Microglia were isolated via the shake-off method. Briefly, flasks were shaken for 2–4 h at 37°C and the resultant detached microglia were spun down and resuspended at desired cell density.

Bone marrow-derived macrophages were prepared from mice by flushing the femurs with 1% FBS in PBS and then cultured in RPMI containing 10% L929 media for 7 days. Resident peritoneal macrophages were collected from mice by flushing the peritoneal cavity with 1% FBS in PBS and then plating collected cells in XVIVO-10 supplemented with 1% PSQ (Pen-Strep-Glut). Cells were allowed to adhere overnight and floaters were washed off; remaining cells were used in the phagocytosis assay a day later.

### MIGRATION ASSAY

Chemoattractants included soluble fractalkine (sFKN; 0.1–10 nM, chemokine domain, R&D systems), CXCL12 (100 ng/mL, R&D systems), and BCM harvested from control or ethanol-treated pups. An anti-fractalkine antibody (3.5 μg/mL, rat monoclonal, R&D systems) was used in some experiments to neutralize fractalkine. Chemokines were prepared in 0.1% BSA in DMEM and BCM was used neat. Chemoattractants were added to 12-well plates at a volume of 600 μL per well and allowed to equilibrate in the incubator for 30 min prior to addition of transwell inserts (Millicell-PCF inserts, 8 um pore size, Millipore) and microglia. Microglia were isolated via the shake-off method, resuspended in 0.1% BSA in DMEM, and 5 × 10^4^ cells were added in 400 μL to the upper chamber, according to transwell instructions. Plates were placed in the incubator for the duration of migration. After 3 h, transwells were placed in 4% PFA with DAPI for 20 min in order to fix cells and stain nuclei. The top of the inserts was wiped clean and only migrated cells remained on the membrane. The membrane was imaged (6–8 fields), and the number of cells per field was averaged for each transwell. Replicates were biological replicates, that is, each replicate data point represents microglia harvested from a different animal.

### CONFOCAL IMAGING

Free-floating sections were cut to a thickness of 40 μm and stained with DAPI. Confocal imaging was performed using a Leica SP5 X. Images in stacks were 0.5 μm apart and the depth collected was 20 μm. Images were acquired from the cortex and data represents the average of 3–6 fields per animal. Microglia were visualized with endogenous GFP expression and apoptotic cells were identified via their DAPI-stained pyknotic nuclei.

### PHAGOCYTOSIS

Thymocytes from 6 to 8 week old mice were incubated with 50 μM of dexamethasone for 4 h and then labeled with CypHer5E. Stained thymocytes were resuspended in glia-conditioned medium and added to phagocytes. The cells were then spun down and incubated at 37°C, 5% CO_2_ for 1 h. After completion of the engulfment assay, the wells were washed three times with PBS, trypsinized, and resuspended in glia-conditioned medium and analyzed by two-color flow cytometry. The microglial cells were recognized by their GFP fluorescence. For each point, 10,000 GFP-positive events were collected and the data was analyzed using FlowJo software.

### QUANTITATIVE PCR

For quantitative PCR using brain tissue, pieces of lateral cortex were isolated and stored at –80°C until RNA isolation. RNA was isolated using RNeasy Lipid Mini kit (Qiagen). Reverse transcription was performed using 1000 ng of RNA according to manufacturer’s instructions (High Capacity cDNA kit, Applied Biosystems). Quantitative PCR was performed with Sybr green according to manufacturer’s instructions (Platinum Sybr kit, Life Technologies) with annealing temperatures of 60°C. Primers used were: actin, forward CCCAGAGCAAGAGAGGTGTC, reverse AGAGCATAGCCCTCGTAGAT; IL-6, forward GAGGATACCACTCCCAACAGACC, reverse AAGTGCATCATCGTTGTTCATACA; TNFα, forward GGCAGGTCTACTTTGGAGTCATTGC, reverse ACATTCGAGGCTCCAGTGAATTCGG; CX3CL1, forward CTCACGAATCCCAGTGGCTT, reverse TTTCTCCTTCGGGTCAGCAC; CX3CR1, forward TGCAGAAGTTCCCTTCCCATC, reverse GGCCTCAGCAGAATCGTCATA; CXCR1eGFP, forward (same as CX3CR1) TGCAGAAGTTCCCTTCCCATC, GFP reverse CTGAACTTGTGGCCGTTTAC. Specificity of CX3CR1, CX3CR1eGFP, and CX3CL1 was confirmed by lack of amplification in respective KO tissues.

## RESULTS

### FRACTALKINE OR CX3CR1 DEFICIENCY LEADS TO AN INCREASE OR PERSISTENCE OF APOPTOTIC DEBRIS IN THE BRAIN AFTER ETHANOL-INDUCED APOPTOSIS

**FIGURE 1:**
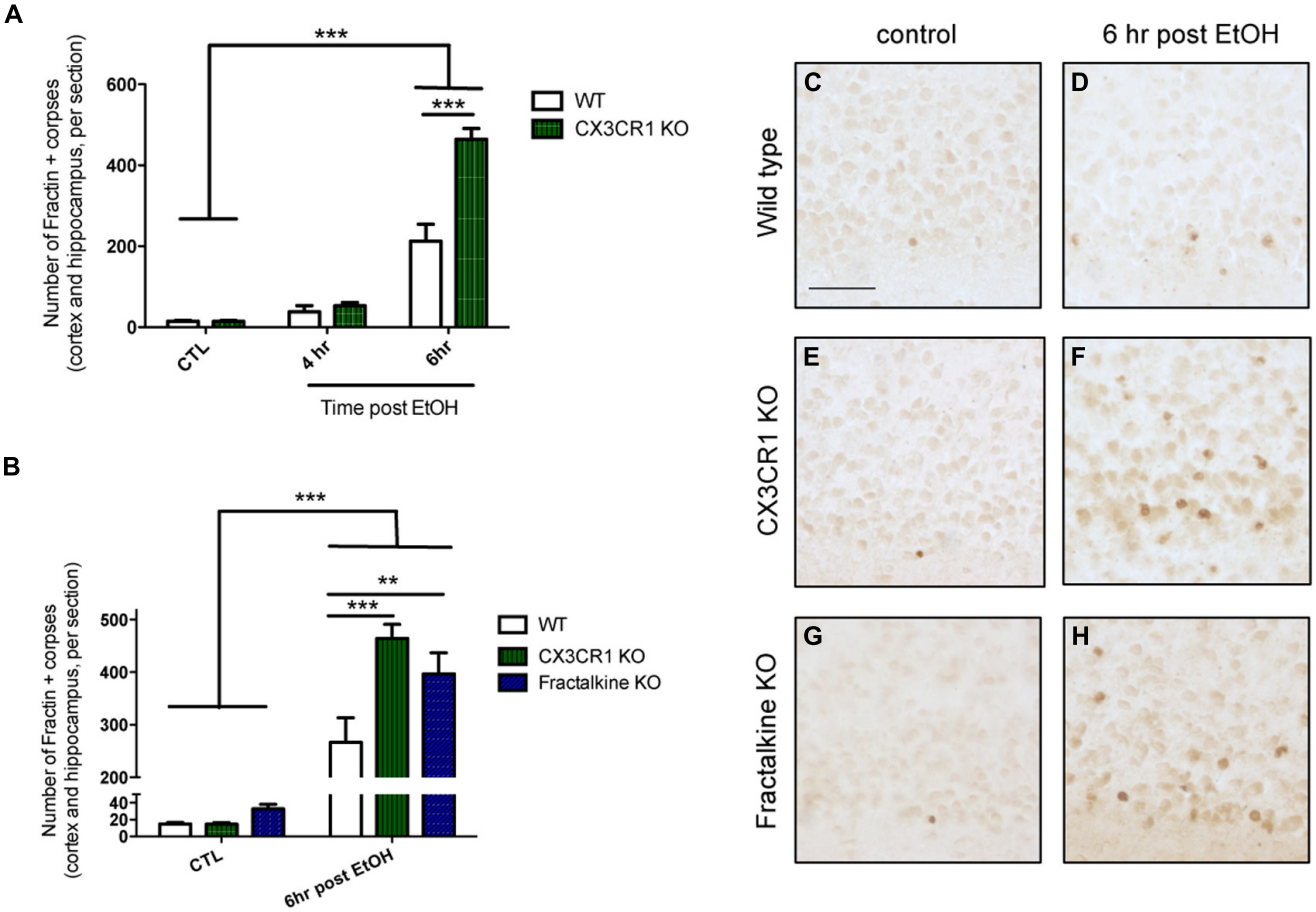
**Deficiency in fractalkine or CX3CR1 leads to increased apoptotic debris. (A)** P7 mice were injected with saline as a control (CTL) or ethanol to induce neuronal apoptosis. Brain tissue was harvested at 4 or 6 h after the initial injection and the number of fractin-positive apoptotic corpses in the cortex and hippocampus was counted (three sections averaged per animal). **(A)** CX3CR1-knockout (KO) and wild type (WT) mice were compared at 4 and 6 h after injection. At 6 h after ethanol injection, the number of fractin-positive corpses was increased in CX3CR1-KO mice compared to WT mice **(B)**. Tissue from WT, CX3CR1-KO, and fractalkine-KO mice was collected 6 h after ethanol injection. CX3CR1-KO and fractalkine-KO mice had increased fractin-positive corpses compared to WT mice. Representative images from the cortex are shown in **(C–H)**. (**A,B**; *n* = 3–6) Two-way ANOVA, **p < 0.01, ***p < 0.005. Scale = 50 μm.

We quantified the amount of apoptotic debris 4 and 6 h after ethanol-induced apoptosis in CX3CR1-KO mice and found that at 6 h the CX3CR1-deficient mice had increased apoptotic debris in the cortex compared to WT mice (**Figures 1A,D,F**). Next, we aimed to determine whether the ligand KO mice phenocopied the receptor KO. We quantified the amount of apoptotic debris 6 h after injury in fractalkine-KO mice, and found that they also had increased apoptotic debris compared to WT animals, and the amount of apoptotic debris was similar to the amount seen in the CX3CR1-KO animals (**Figures 1B,F,H**). Of note, there was no difference between genotypes in the amount of apoptotic corpses in control, uninjured animals (**Figures 1B,C,E,G**). CX3CR1-heterozygous (HET) mice (which were used for subsequent experiments) had levels of debris comparable to WT animals (Figure S1).

### *IN VIVO* ETHANOL-INDUCED NEURONAL APOPTOSIS LEADS TO RELEASE OF SOLUBLE FRACTALKINE

**FIGURE 2:**
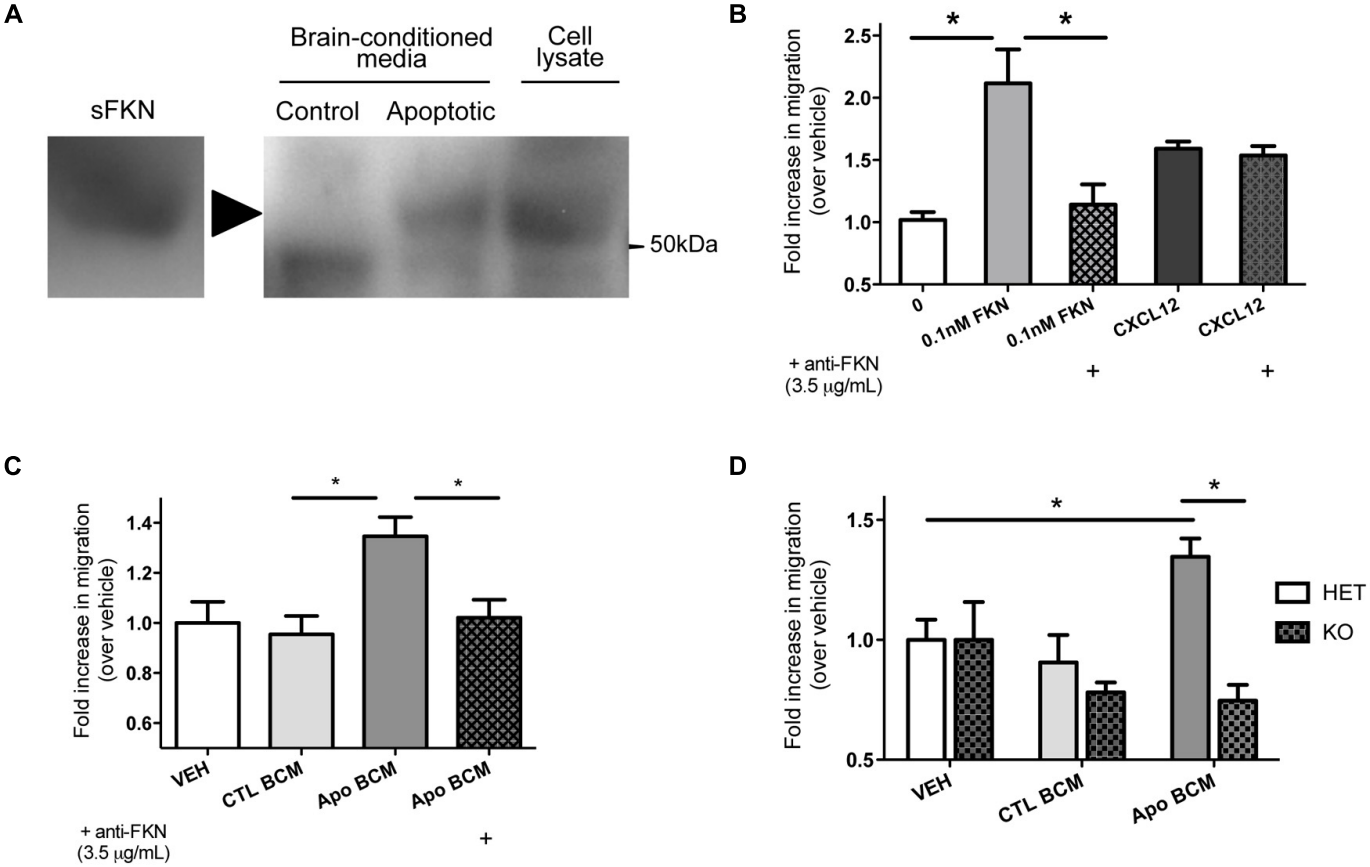
**Soluble fractalkine (sFKN) is released into the extracellular space after injury and acts as a chemotactic factor for microglia. (A)** P7 mice were treated with saline (control) or ethanol to induce neuronal apoptosis (apoptotic), and the brains were incubated in DMEM on ice for 2 h to isolate brain-conditioned media (BCM). 2 mg of protein were TCA precipitated, run on a gel, and probed with anti-fractalkine via western blot. sFKN and lysate from mixed neural cultures were used for comparison. We found fractalkine in BCM from apoptotic, but not control brain (a, arrowhead points to band of interest). **(B–D)** Transwell migration assays were performed to determine whether microglia transmigrate toward fractalkine. Microglia were isolated from mixed glial cultures via the shake-off method and added to the upper chamber of a transwell insert. Attractant of interest was added to the bottom of the transwell. After 3 h of migration, the cells that had migrated to the bottom surface of the transwell were fixed, stained with DAPI, and counted. **(B)** Microglia migrate toward 0.1 nM of sFKN. A fractalkine-neutralizingantibody (3.5 μg/mL) was pre-incubated with either 0.1 nM of sFKN or CXCL12 [indicated by (+)]. This antibody specifically blocked fractalkine-induced migration, as CXCL12-induced migration was not inhibited. **(C,D)** Microglia migrate toward apoptotic BCM in a fractalkine-dependent manner. P7 mice were treated with saline as a control or ethanol to induce neuronal apoptosis. Brains were then incubated in DMEM on ice for 2 h to isolate BCM from saline-treated control (CTL BCM) or apoptotic brain (Apo BCM). BCM was used as a chemoattractant in the lower chamber for transwell migration assays. **(C)** Microglia migrate toward apoptotic BCM but not control BCM. A fractalkine-neutralizing antibody (3.5 μg/mL) was pre-incubated with apoptotic BCM [indicated by (+)] to block fractalkine-dependent migration and this prevented migration toward the apoptotic BCM. **(D)** Migration toward BCM was quantified in CX3CR1 heterozygous (HET) versus KO microglia. CX3CR1 deficient microglia fail to migrate toward apoptotic BCM. **(B–D)** Data is from repeated experiments, each replicate represents microglia harvested from a different animal (*n* = 4–6). **(B–C)** One-way ANOVA, *p < 0.05; **(D)** Two-way ANOVA, *p < 0.05.

We observed increased apoptotic debris in mice that are deficient in fractalkine signaling, which suggests fractalkine plays an important role in the response to alcohol injury. We hypothesized that the cleaved fragment of fractalkine is released during ethanol-induced apoptosis. We harvested brains 6 h after ethanol-induced apoptosis and incubated them in media to isolate diffusible extracellular factors and tested whether fractalkine was present. We detected sFKN in BCM from animals treated with ethanol, but not in saline-treated controls (**Figure 2A**). This shows that fractalkine is released as a soluble fragment in response to ethanol-induced injury. Recombinant fractalkine and lysate from mixed neural cultures were used as controls for the western blot.

### MICROGLIA MIGRATE TOWARD APOPTOTIC BRAIN-CONDITIONED MEDIA IN A FRACTALKINE AND CX3CR1-DEPENDENT MANNER

We hypothesized that fractalkine released during injury plays a role in the microglial reaction to apoptotic neurons. Microglia are the only cells in the brain that express appreciable levels of the fractalkine receptor, CX3CR1. Fractalkine is a known chemotactic factor, and we aimed to determine whether fractalkine released from apoptotic neural cells was necessary and sufficient to induce microglial chemotaxis.

We found that microglia migrate toward sFKN; migration toward fractalkine was dose-dependent, and as expected, CX3CR1-KO microglia did not migrate toward fractalkine (Figure S2). CX3CR1 deficiency does not lead to a general migration defect as KO microglia are still able to migrate toward another chemokine, CXCL12 (Figure S2). A fractalkine-neutralizing antibody blocks migration toward fractalkine, and this antibody is not a general inhibitor of migration, as it has no effect on migration toward CXCL12 (**Figure 2B**).

Next, we tested whether microglia respond to the fractalkine in the extracellular media from apoptotic brains. The apoptotic BCM from ethanol-treated animals was sufficient to induce migration (**Figure 2C**). Microglia did not migrate toward the BCM from the non-apoptotic control tissue. Fractalkine signaling was absolutely required to stimulate migration toward the apoptotic BCM. Neutralizing fractalkine blocked migration toward the apoptotic BCM (**Figure 2C**), and CX3CR1-deficient microglia failed to migrate toward it (**Figure 2D**). This data suggests that in the context of ethanol-induced injury, sFKN signals to microglia in a CX3CR1-dependent manner. Our data suggests that this signaling helps attract microglia toward apoptotic neurons.

### *IN VIVO* ANALYSIS OF PROXIMITY BETWEEN MICROGLIA AND APOPTOTIC NEURONS

**FIGURE 3:**
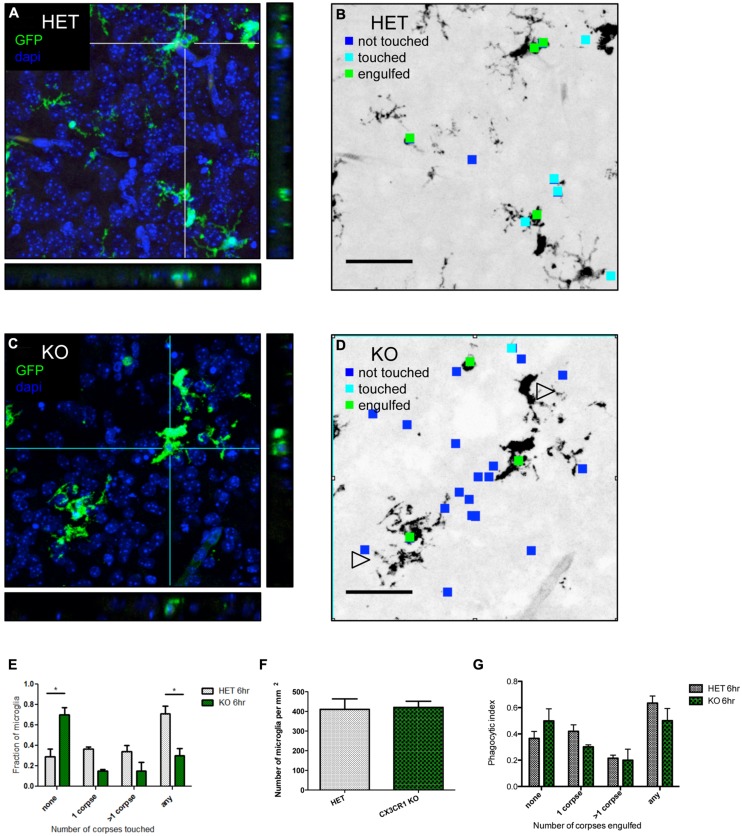
**CX3CR1-deficient microglia have a defect in association with apoptotic debris after ethanol-induced apoptosis, but no overt defect in phagocytosis. (A–D)** CX3CR1-HET and CX3CR1-KO pups were injected with ethanol to induce neuronal apoptosis and tissue was harvested 6 h later. Microglia express GFP and nuclei were labeled with DAPI. 40 μm sections were cut and 20 μm thick confocal stacks were acquired from the cortex. Apoptotic corpses were identified via their pyknotic nuclei. **(A,C)** Representative images with orthogonal projections acquired from CX3CR1-HET **(A)** and CX3CR1-KO **(C)** after ethanol treatment. **(B,D)** We analyzed each confocal stack and identified apoptotic cells as either untouched (blue square), touched (cyan square) or engulfed (green square) by microglia. Open arrowheads in **(D)** indicate microglial processes that fail to associate with nearby apoptotic corpses. **(E)** An association index was determined by quantifying the fraction of microglia touching apoptotic corpses at 6 h after injury. By 6 h, fewer microglia from the CX3CR1-KO had associated with apoptotic corpses as compared to the CX3CR1-HET microglia. **(F)** The density of microglia was quantified and there was no difference between the CX3CR1-HET and CX3CR1-KO. **(G)** Confocal stacks were also used to calculate a phagocytic index, the fraction of GFP-positive microglia that contained corpses. There was no significant difference in phagocytosis measures in the brain of the CX3CR1-KO compared to the CX3CR1-HET mice. (**E–G**; *n* = 3, three fields were averaged per animal) Two-way ANOVA, *p < 0.05. Scale = 50 μm.

We hypothesized that fractalkine signaling modulates microglial recruitment to apoptotic cells. Brain tissue from CX3CR1-KO or CX3CR1-HET pups was collected at 6 h after ethanol treatment and confocal stacks were acquired (orthogonal views seen in **Figures 3A,C**). Microglia were identified via endogenous GFP expression and apoptotic cells were identified via their pyknotic, DAPI-positive nuclei. We analyzed the confocal stacks and identified apoptotic cells as either untouched, touched or engulfed by microglia (**Figures 3B,D** are diagrams depicting the analysis for **Figures 3A,C**). An association index was calculated by quantifying the fraction of microglia touching apoptotic cell bodies (arrowheads in **Figure 3D** indicate microglial processes that fail to associate with nearby apoptotic corpses). CX3CR1-KO microglia had a lower association index at 6 h after injury (**Figure 3E**).

There was no difference in microglial cell density between the HET or the KO animals (**Figure 3F**). Therefore the increase in debris in the CX3CR1-KO brain is not attributable to a difference in number of microglia available to participate in clearance. Microglia are normally ubiquitous throughout brain tissue, therefore long distance migration may not be required for the microglial response. Instead, fractalkine may signal for local “recruitment” of microglia, and this could manifest as movement of just the arms or processes of the cell as opposed to the entire cell.

An increase in apoptotic debris could be due to defects in microglial recruitment, but could also be due to a defect in microglial phagocytosis and clearance. We used the same confocal images to determine whether CX3CR1-deficient microglia had an *in vivo* defect in phagocytosis. The microglial phagocytic index was quantified by measuring the fraction of microglia containing engulfed apoptotic cells (**Figure 3G**). We found no change in the phagocytic index at 6 h after ethanol treatment.

We also performed *in vitro* phagocytosis assays to determine whether fractalkine signaling modulates phagocytosis. We tested whether sFKN promotes phagocytosis and whether CX3CR1-KO cells have a defect in phagocytosis. We utilized multiple approaches. We used apoptotic thymocytes as targets and tested macrophages pretreated with fractalkine or treated concurrent with addition of apoptotic cells (Figure S3). Addition of exogenous fractalkine had no effect and CX3CR1-deficient macrophages had a comparable phagocytic index. Next, we tried pure cultures of microglia isolated from CX3CR1 HET or KO glial cultures, but found no effect of fractalkine or CX3CR1 deficiency (Figure S3). Finally, we tried mixed cultures of astrocytes and microglia. We added apoptotic thymocytes to mixed glial cultures from CX3CR1 HET or KO animals treated with or without fractalkine and quantified the microglial phagocytic index and found that neither exogenous fractalkine nor CX3CR1-deficiency had an effect (Figure S3). The combination of *in vitro* and *in vivo* data suggest that fractalkine signaling does not have a prominent role in regulating the engulfment phase of phagocytosis.

We have not ruled out the possibility that CX3CR1-KO microglia have a defect in their ability to digest apoptotic cells, which could also lead to a persistence of debris after ethanol-induced apoptosis. It is also possible that CX3CR1 deficiency leads to increased apoptotic debris through a combination of decreased clearance and increased neurotoxicity.

### FRACTALKINE OR CX3CR1 DEFICIENCY LEADS TO AN ALTERED INFLAMMATORY GENE EXPRESSION RESPONSE TO ETHANOL-INDUCED APOPTOSIS

**FIGURE 4:**
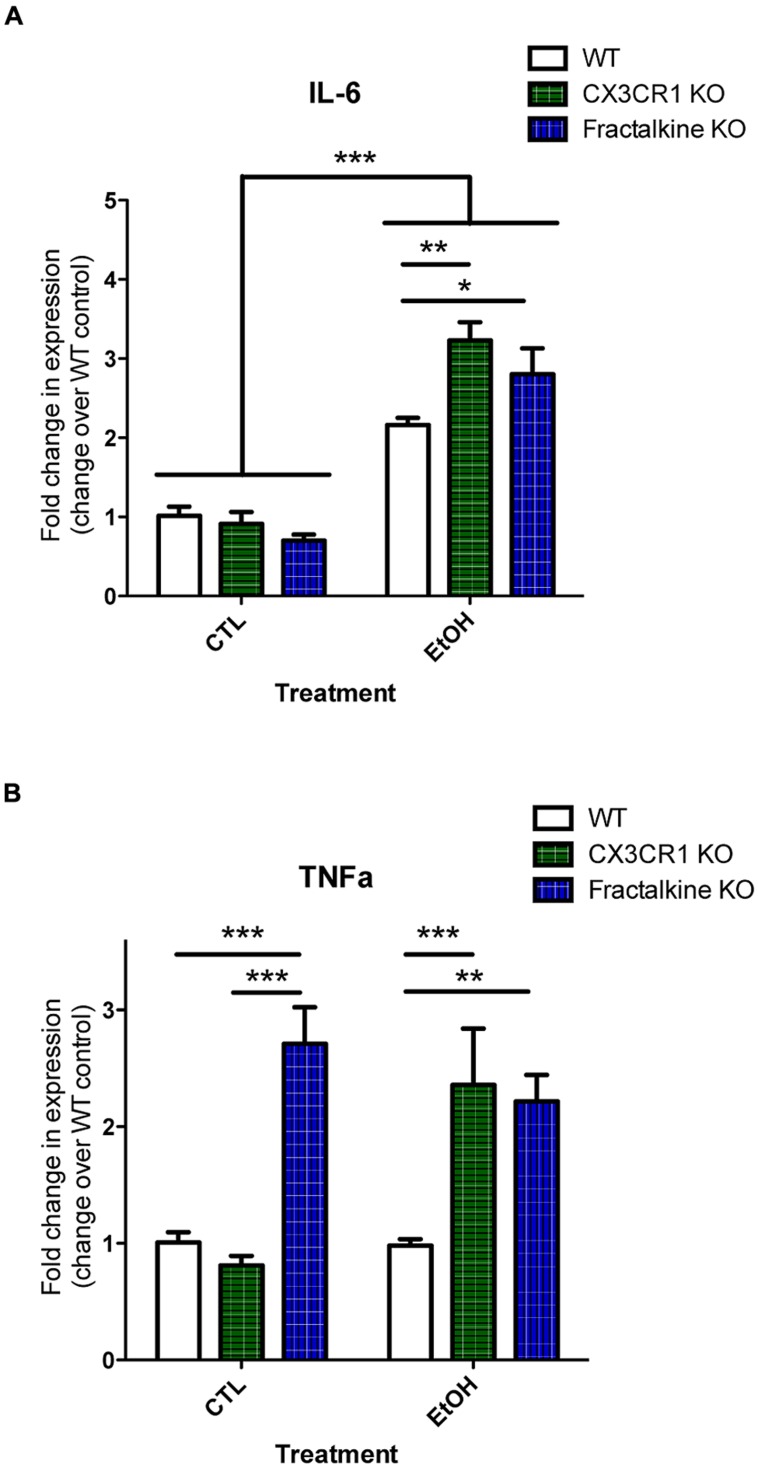
**Ethanol-induced apoptosis leads to fractalkine-dependent alteration of cytokines gene expression.** P7 animals were treated with saline (CTL) or ethanol and brain tissue was harvested 6 h later. Brain tissue and RNA was harvested from WT, CX3CR1-knockout, and fractalkine-knockout mice. Quantitative PCR was used to assess mRNA expression of IL-6 **(A)** and TNAα **(B)**. IL-6 expression is elevated after ethanol treatment, and is significantly higher in the CX3CR1 knockout and the fractalkine-knockout than in the wild type brain. In fractalkine-knockout brain, TNFα expression is higher in control conditions as compared to wild type or CX3CR1 knockout. CX3CR1-knockout brain has increased TNFα compared to wild type after ethanol treatment. (*n* = 3–6; **A,B**) Two-way ANOVA, *p < 0.05, **p < 0.01, ***p < 0.001.

Apoptotic cells and fractalkine are both known to modulate inflammatory responses. Therefore, we hypothesized that the increased apoptotic load and/or defective fractalkine signaling would lead to an altered inflammatory response in the brain after ethanol injury. We quantified mRNA expression of the cytokines IL-6 and TNFα as well as fractalkine and CX3CR1 (**Figure 4**; Figure S4). IL-6 levels were similar in control-treated animals of each genotype. In WT animals, we found increased IL-6 mRNA at 6 h after ethanol treatment. IL-6 expression was further increased in both fractalkine- and CX3CR1-KO mice compared to WT. TNFα levels were similar in control-treated WT and CX3CR1 mice, but only CX3CR1-KOs upregulated TNFα at 6 h after ethanol injury. In contrast, fractalkine-KO mice had increased TNFα expression at baseline, at levels comparable to the levels seen in ethanol-treated CX3CR1 mice, and the level did not change with ethanol treatment (**Figure 4**).

We also tested whether fractalkine or CX3CR1 expression was modulated by ethanol-induced apoptosis (Figure S3). We found no difference in mRNA expression in the brain of WT animals after ethanol treatment compared to control. We also analyzed expression in the KO animals. We found an increase in fractalkine mRNA expression in the CX3CR1-KO animals after ethanol treatment. We assessed expression of transcript for the receptor and we found increased CX3CR1 in the fractalkine-KO. The CX3CR1-KO does not express functional CX3CR1, however, GFP transcript can be used as a reporter for gene expression. We used a primer set designed to amplify CX3CR1-eGFP transcript and we found increased production of this transcript in the CX3CR1-KO after ethanol treatment. This suggests that fractalkine signaling could have a role in feedback regulation of the expression of the receptor and ligand in the context of injury.

## DISCUSSION

Fractalkine has been previously described as a neuron–microglia communication factor and is known to be a chemokine that can modulate migration of immune cells (Ransohoff et al., 2007). Our data additionally illuminates the role of fractalkine signaling in the response to apoptotic neuronal cells in the context of perinatal brain injury.

Only one previous study has investigated fractalkine regulation after ethanol injury. In that study, the quantity of fractalkine protein increased after prenatal ethanol injury in WT mice (Roberson et al., 2011). Using our model, we did not detect changes in fractalkine mRNA expression in WT mice after postnatal ethanol injury (Figure S3). However, when we isolated BCM, that is, the extracellular soluble components from the brain, we detected an increase in cleaved sFKN after ethanol injury (**Figure 2A**).

Cleaved fractalkine released from apoptotic neurons may create a gradient that allows microglia to hone in on apoptotic neurons. A previous study showed that fractalkine acts as a “find me” signal to guide phagocytes such as macrophages to apoptotic cells (Truman et al., 2008), and others have shown that fractalkine can modulate chemotaxis of microglia (Maciejewski-Lenoir et al., 1999). However, these studies predominantly used *in vitro* models or peripheral systems. We show that fractalkine acts as an important “find me” signal to modulate microglial recruitment in order to help clear apoptotic neurons in *in vivo* CNS injury.

Apoptosis may result in the release of many factors, and fractalkine is only one of many possible chemoattractants (Ravichandran, 2003; Ravichandran and Lorenz, 2007). However, we show that fractalkine signaling is critical for migration toward apoptotic BCM: blocking fractalkine signaling by neutralizing fractalkine or through CX3CR1 deficiency prevents migration (**Figure 2**). Does this suggest other “find me” signals are irrelevant?

Other signals such as ATP and UDP have been shown to modulate microglial movement and phagocytosis (Davalos et al., 2005; Koizumi et al., 2007). ATP and UDP are unstable in the extracellular space due to the presence of ubiquitous ATPases (Zimmermann, 2000). It seems possible that fractalkine may have a longer half-life than other potential “find-me” signals and therefore fractalkine may be the dominant chemoattractant remaining in apoptotic BCM. These other signals may still be relevant in other conditions.

Our *in vitro* data suggests that fractalkine “find me” signaling is the critical factor for recruitment of microglia; however, *in vivo*, other signals are probably also involved. Although we found that microglia from CX3CR1-KO mice did not associate with apoptotic debris as well as WT microglia (**Figure 3**), there was not a large *in vivo* defect in engulfment (Figure S2). This suggests two possibilities: (a) microglia are ubiquitous and motile enough that in the absence of fractalkine signaling they are still able to encounter apoptotic debris by chance and engulf it, or (b) perhaps there are other factors that can act to recruit microglia in the absence of fractalkine signaling. For example cell surface “eat me” signals are likely involved.

A defect in recruitment or the association of microglia with apoptotic neurons would lead to failed or slowed clearance of debris. We speculate that this explains why the fractalkine and CX3CR1-KO brains have more apoptotic debris after ethanol injury compared to WT animals (**Figure 1**).

The increased apoptotic debris could be attributable to a defect in recruitment, but could also be due to a defect in phagocytosis. A previous study showed that addition of sFKN to injured cultured neurons induces phagocytosis (Noda et al., 2011). However, we could not find any evidence for an *in vivo* defect in phagocytosis, as the microglial phagocytic index was similar in WT and CX3CR1-KO animals (**Figure 3**). In addition, we tested *in vitro* phagocytosis and found no effect of fractalkine and no defect in the CX3CR1-deficient microglia (Figure S2). Another factor that would influence the amount of debris is the rate of degradation of apoptotic material, and we have not ruled out the possibility that CX3CR1-KO microglia could have a defect in the ability to digest corpses. This could be tested by following the fate of apoptotic cells in time lapse imaging experiments both *in vitro* and perhaps *in vivo* using slice cultures of alcohol-injured brain.

We do not know if other glia also plays a role in clearance of apoptotic cells. Astrocytes are capable of engulfment (Noda et al., 2011), and it is possible that they may engulf debris after alcohol injury. However, we were unable to identify parenchymal astrocytes via conventional astrocyte markers such as glial fibrillary aidic protein (GFAP) or S100β at this age in development. Therefore, we were unable to determine whether astrocytic engulfment contributed to clearance. Astrocytes do not express appreciable levels of CX3CR1 *in vivo*; therefore they are unlikely to be responding to fractalkine in this injury.

A common dogma in the field of apoptotic cell clearance is that cells must be cleared in order to prevent an exaggerated immune response (Ravichandran and Lorenz, 2007). Interestingly, we find that in conjunction with the increased apoptotic cell load, we see an exaggerated immune response in the fractalkine and CX3CR1-KO animals. It would be interesting if this exaggerated inflammatory response were due to persistence of apoptotic debris and secondary necrosis. However, we cannot tease apart whether this altered immune response is due the persistence of apoptotic cells or due to the defect in fractalkine signaling, as both apoptotic cells and fractalkine have been shown to modulate inflammatory responses (Mizuno et al., 2003; Griffiths et al., 2009). Related to this, another possibility we have not ruled out is that the increase in apoptotic debris could be due to increased neurotoxicity because of an adverse inflammatory response. Fractalkine may be important for immunomodulation (perhaps suppression). Fractalkine has been shown to downregulate production of pro-inflammatory factors in response to LPS (Mizuno et al., 2003). Avoiding the exaggerated immune response may be critical to avoid secondary degeneration.

Another goal of this study was to determine whether there is an inflammatory response after acute ethanol injury. Our data supports previous data that showed that fetal alcohol injury leads to an increase in factors such as TNFα, and IL-6 (Vink et al., 2005). We similarly find that ethanol injury induces an inflammatory response in the developing brain. We additionally show that this response appears to be modulated by fractalkine signaling. Ultimately, *in vivo* we cannot tease apart whether the ethanol or the apoptotic cells are signaling for the production of cytokines, and we do not know the downstream functional consequences of this inflammation.

It is interesting that the ligand and receptor KO phenocopied with respect to increased apoptotic debris after injury, but had differences in mRNA expression of TNFα. It is possible that intact fractalkine transmembrane protein has other important functions distinct from its role in CX3CR1-receptor signaling that would explain baseline (pre-injury) differences in TNFα expression in the fractalkine-KO brain. Fractalkine is known as an adhesion molecule. Perhaps it also associates with and modulates other signaling molecules or has an (as of yet unidentified) intracellular signaling component. It is also interesting that fractalkine and TNFα are both cleaved by ADAM 17 (also known as the TNFα-converting enzyme, TACE). Perhaps fractalkine deficiency somehow leads to dysregulation of TNFα due to this shared relationship with ADAM 17.

## CONCLUSION

We have used a well-characterized mouse model of ethanol-induced apoptosis to assess the role of fractalkine in neuronal–microglial signaling. Our data suggests that fractalkine released after apoptosis recruits microglial processes toward apoptotic cells to promote their clearance and that defects in this signaling lead to increased apoptotic debris. Secondly, our data suggests that defects in clearance or fractalkine signaling lead to altered cytokine production after ethanol injury. Collectively, this suggests that fractalkine acts as a “find me” signal released by apoptotic neurons and subsequently plays a critical role in modulating clearance and inflammatory cytokine gene expression after ethanol-induced apoptosis.

## AUTHOR CONTRIBUTIONS

Jennifer D. Sokolowski and James W. Mandell conceived and designed the project. Jennifer D. Sokolowski, Chloe N. Chabanon-Hicks, Claudia Z. Han, and Daniel S. Heffron performed the experiments. Jennifer D. Sokolowski prepared all the figures and wrote the manuscript with assistance from Chloe N. Chabanon-Hicks. All authors reviewed the manuscript.

## Conflict of Interest Statement

The authors declare that the research was conducted in the absence of any commercial or financial relationships that could be construed as a potential conflict of interest.

## Supplementary material

The Supplementary Material for this article can be found online at:
