# Supplementary material for: Fractalkine is a “find-me” signal released by neurons undergoing ethanol-induced apoptosis
Source: Front Cell Neurosci. 2014 Nov 7;8:360. doi: 10.3389/fncel.2014.00360 (PMC4224129; doi:10.3389/fncel.2014.00360)
Supplement: Supplementary file 1 [file Data_Sheet_1.PDF]

Supplementary Material

Fractalkine is a "find-me" signal released by neurons undergoing ethanol-induced apoptosis

Jennifer D. Sokolowski*[123], Chloe Chabanon-Hicks[1], Claudia Han[4], Daniel S. Heffron[1], James W. Mandell[1].

[1] Department of Pathology, University of Virginia School of Medicine, Charlottesville, VA 22908
[2] Medical Scientist Training Program, University of Virginia School of Medicine, Charlottesville, VA 22908
[3] Neuroscience Graduate Program, University of Virginia School of Medicine, Charlottesville, VA 22908
[4] Department of Microbiology, Immunology and Cancer Biology, University of Virginia School of Medicine, Charlottesville, VA 22908

- **Correspondence:** . Jennifer Sokolowski, 415 Lane Rd, MR5 3220, Charlottesville, VA 22908, USA. jde2z@virginia.edu

1. Supplementary Figures

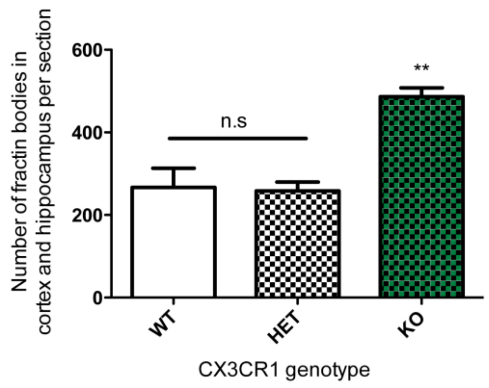

Figure S1. There was no significant difference in debris between CX3CR1-heterozygous animals (HET) and knockout animals at 6 hours after ethanol treatment, justifying the use of heterozygous animals as controls for future experiments **(B)**. One-way ANOVA, (n=3-6) **p<0.01.

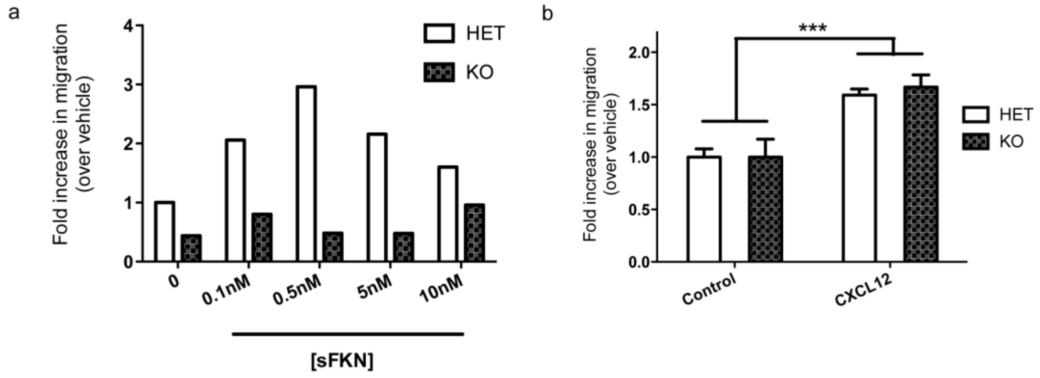

Figure S2. Transwell migration assays were performed to determine whether microglia transmigrate toward the chemoattractants fractalkine and CXCL12. Microglia were isolated from mixed glial cultures via the shake-off method and added to the upper chamber of a transwell insert. Attractant of interest was added to the bottom of the transwell. After 3 hours of migration, the cells that had migrated to the bottom surface of the transwell were fixed, stained with DAPI, and counted. **(A)** A dose-response curve was performed using CX3CR1-heterozygous (HET) and CX3CR1-knockout (KO) microglia to measure attraction toward soluble fractalkine concentrations ([sFKN]) ranging from 0.1nM to 10nM. This showed that 0.1nM was sufficient to induce migration and that CX3CR1-knockout microglia fail to migrate toward fractalkine. Fold change in migration was calculated compared to migration of heterozygous microglia toward vehicle. **(B)** CX3CR1-knockout microglia were tested for a general migration defect using CXCL12 (100ng/mL) as a chemoattractant. CX3CR1-heterozygous and knockout microglia migrated toward CXCL12 to a comparable degree. (A) (n=1) (B) Data is from repeated experiments, each replicate represents microglia harvested from a different animal (n=4-6). (B) 2-way ANOVA, ***p<0.005.

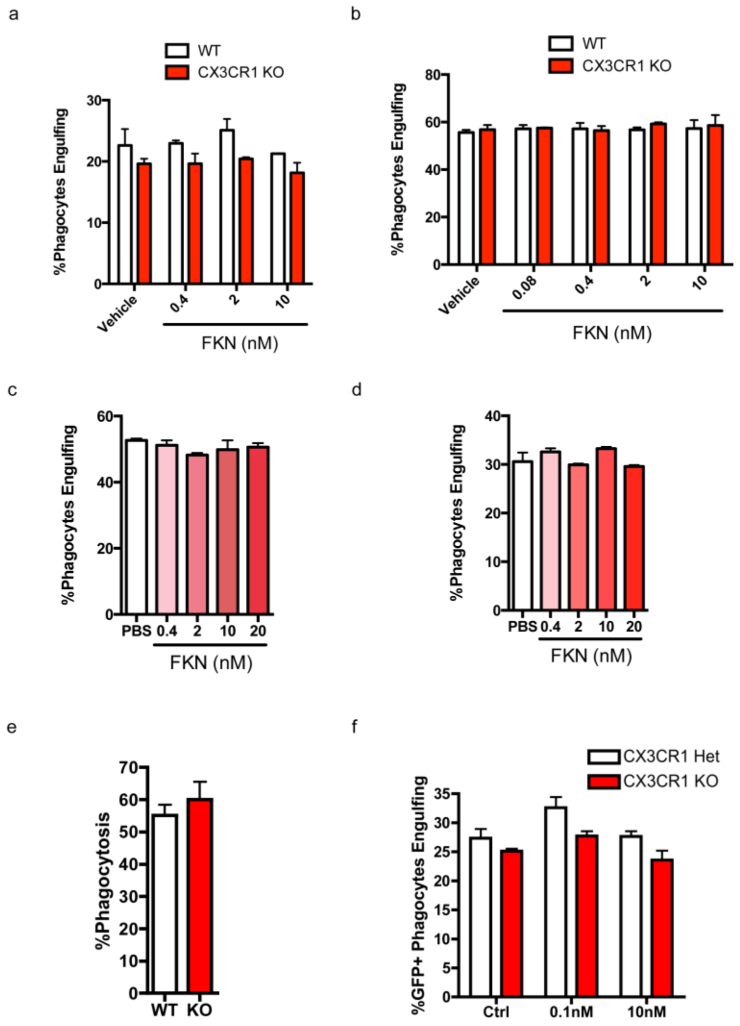

Figure S3. CX3CR1-deficient phagocytes do not have an overt defect in phagocytosis *in vitro*. Apoptotic thymocytes were used as targets, and added to either macrophages or glia and phagocytosis was allowed to proceed for 1 hour. Phagocytosis was measured via flow cytometry and the phagocytic index (fraction of cells engulfing) was calculated. Wild type (WT) and CX3CR1-knockout (KO) bone-marrow derived macrophages were treated concurrent with administration of apoptotic cells **(A)** or pretreated for 20 hours **(B)** with soluble fractalkine (s-FKN) at indicated concentrations. Wild type and CX3CR1-knockout bone-marrow derived macrophages **(C)** and peritoneal macrophages **(D)** were pretreated for 24 hours with the indicated concentrations of soluble fractalkine (FKN) before the phagocytosis assay. A phagocytosis assay was also performed on microglia isolated from wild type and CX3CR1-knockout mixed glia cultures **(E)**. CX3CR1-heterozygous and CX3CR1-knockout mixed glial cultures were incubated with 0 (Ctrl), 0.1nM and 10nM of soluble fractalkine and the microglial phagocytic index was quantified by gating on GFP-positive cells **(F)**. We detected no difference in phagocytosis with addition of exogenous fractalkine and did not detect a phagocytic defect in CX3CR1-knockout cells.

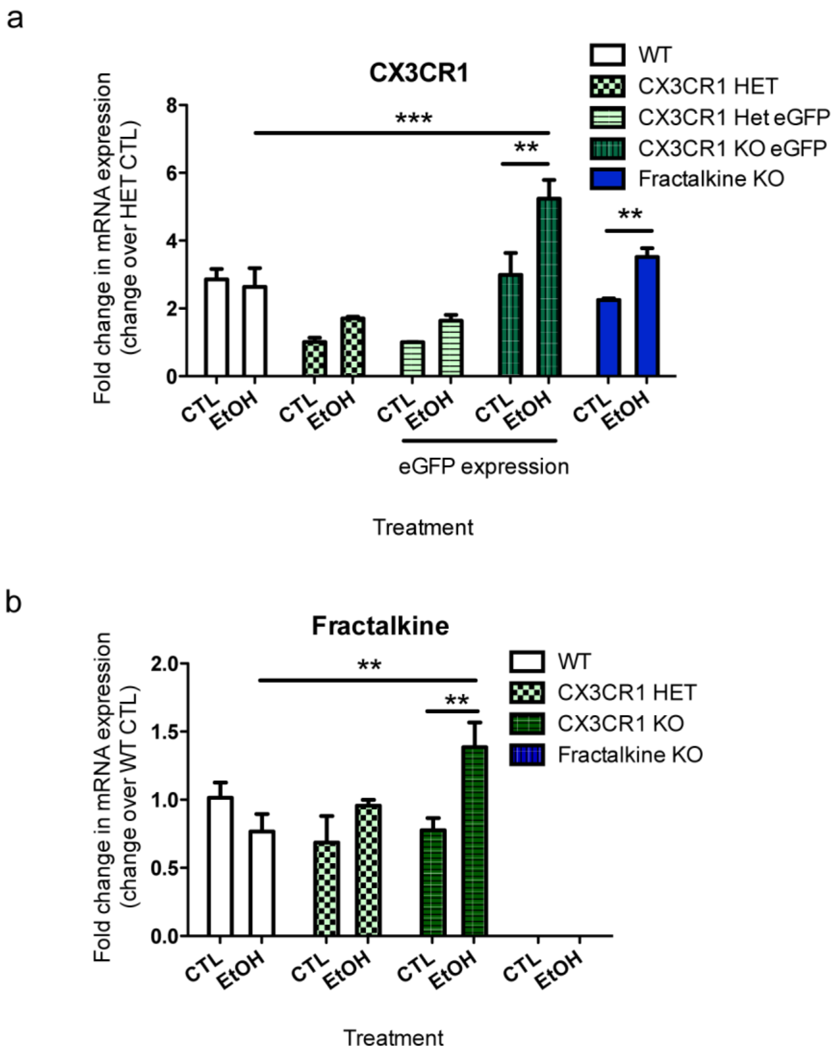

Figure S4. Fractalkine and CX3CR1eGPP mRNA is increased in CX3CR1-knockout mice after ethanol injury. P7 animals were treated with saline (CTL) or ethanol (EtOH) and brain tissue was harvested 6 hours later. RNA was isolated and quantitative PCR was used to assess mRNA expression. Wild type (WT), CX3CR1 heterozygous, CX3CR1-knockout, and fractalkine-knockout brain tissue was probed for mRNA expression of CX3CR1 or the GFP reporter **(A)** and fractalkine **(B)**. In order to relate receptor expression and compare wild type to CX3CR1-deficient mice, we used two sets of primers. One set amplified CX3CR1 and the other amplified the GFP transcript expressed in place of the gene in the CX3CR1-knockout allele. CX3CR1 expression was normalized to control-treated heterozygous mice, which have a copy of the wild type allele and a copy of the eGFP reporter (which is the knockout allele) **(A)**. Fractalkine expression was normalized to control-treated wild type mice **(B)**. Fractalkine-knockout mice and CX3CR1-knockout mice had increased expression of the CX3CR1 gene after ethanol treatment. Fractalkine expression was increased in the CX3CR1 knockout after ethanol treatment. **(A,B)** (n=3-6) 2-way ANOVA, $**p<0.01$, $***p<0.001$.
